# Population Structure of Curraleiro Pé-Duro Cattle and its Relationship With the Serological Profile Against Pathogens of Economic and Zoonotic Interest

**DOI:** 10.3389/fgene.2022.872660

**Published:** 2022-05-13

**Authors:** Thais Miranda Silva Freitas, Juliana Moraes Dias, Ana Carolina Ferreira Veríssimo, Joyce Rodrigues Lobo, Gustavo Lage Costa, Maria Ivete de Moura, Vincenzo Landi, Amparo Martínez Martínez, Adriana Santana do Carmo, Maria Clorinda Soares Fioravanti

**Affiliations:** ^1^ Department of Veterinary Medicine, School of Veterinary and Animal Science, Federal University of Goiás, Goiânia, Brazil; ^2^ Escola de Ciências Médicas e da Vida da Pontifícia Universidade Católica de Goiás, Goiânia, Brazil; ^3^ Università Degli Studi di Bari Aldo Moro, Bari, Italy; ^4^ Department of Genetics, Campus of International Agrifood Excellence, Universidad de Córdoba, Cordoba, Spain; ^5^ Department of Animal Science Medicine, School of Veterinary and Animal Science School, Federal University of Goiás, Goiânia, Brazil

**Keywords:** conservation genetics, infectious diseases, microsatellite, breed characterization, bovine

## Abstract

Curraleiro Pé-Duro (CPD) cattle, a Brazilian local breed, are animals that are highly resistant to infectious and parasitic diseases. Strategies for the conservation of the breed and the genetic resistance to diseases should also consider the characteristics of the breed. The objective of this study was to analyze the diversity and population structure of the CPD breed using microsatellite markers and to correlate the serological profiles for causative agents of brucellosis, leptospirosis, neosporosis, leukosis, infectious bovine rhinotracheitis, and bovine viral diarrhea. DNA samples of 608 bovines were amplified and genotyped using 28 microsatellite markers for breed characterization. The genotypes were assigned to three clusters, indicating a substructure population related to the geographic distance. The observed heterozygosity was lower than that expected in most loci, and fixation index (Fst) in the subpopulation values ranged from 0.03073 (ETH3) to 0.402 (INRA63) on cluster 1, from 0.00 (INRA32) to 0.39359 (INRA63) on cluster 2, and from 0.00 (INRA32) to 0.28483 (TGLA53) on cluster 3. The *Brucella spp*., *Neospora caninum*, and Bovine Leukemia Virus frequencies were significantly different (*p* < 0.05) between clusters. The differences between the occurrences of diseases were not sufficient to indicate a genetic subpopulation with increased resistance to infections.

## Introduction

Local breeds have become a target of interest owing to their ability to live and reproduce in extreme climatic conditions and their adaptation characteristics to local conditions, characterizing them as resilient breeds. These characteristics mean that these breeds have a genetic heritage that should be preserved, especially considering the need for food production in the face of global climate change (FAO, 2011).

During the colonization period, the first cattle were introduced in Brazil from the Iberian Peninsula, which underwent natural selection. They eventually adapted to local conditions and became rustic cattle, tolerant to heat, water, and food stress, with high prolificacy and resistance to diseases and parasites (Fioravanti et al., 2008; Egito et al., 2016). Such animals were not divided into breeds; with the miscegenation and expansion of herds in Brazilian biomes, which have unique characteristics, local Brazilian breeds became differentiated (Egito et al., 2007), although with similar alleles because of their common origin (Martínez et al., 2012).

To prevent commercial breeds from replacing the naturalized Brazilian breed Curraleiro Pé-Duro (CPD), companies and research institutions had initiated plans for the conservation of animal genetic resources. Such initiatives aim to identify populations at risk, characterize them phenotypically and genetically, and evaluate their productive potential, both at *in situ* conservation centers in the central-west and northeast regions of the country and *ex-situ* (Albuquerque et al., 2002; Juliano et al., 2011).

There has been concern about the genetic diversity of local breeds, which could be seriously affected, putting these breeds at risk of extinction. The study of this diversity through geographic genetics aids our understanding of the gene flow and the adaptation of species within heterogeneous and fragmented spaces by understanding the geographical distances and how the transmission of genes occurs, making it possible to detect environmental factors that stimulate the adaptation process (Manel and Holderegger 2013).

Animals separated by geographic distances may present different genetic structures, to the extent of leading to subdivisions between breeds and within the same breed (Souza et al., 2022). Geographic isolation, reduced number of individuals, and adaptation to different environments can generate genetic drift, changing the frequency of alleles accumulated over generations. Therefore, genetic characterization allows the identification of groups that were isolated in the environment for a long time and became genetically distinct groups (Egito et al., 2007; 2016).

Genetic characterization is a criterion used to decide which population should be conserved and is especially important to optimize the choice of samples when resources are scarce, ensuring genetic variability. The selection of breeds and individuals that should participate in the conservation program considers economic interests, adaptability, and the presence of unique alleles (Mariante et al., 2009).

The adaptive characteristics of animal genetic resources can be transmitted to other generations. Therefore, the most adapted genotypes need to be identified and managed. The purpose of identifying genotypes and preserving local breeds is for the possible future use of genetic material as a way to introduce their unique characteristics of adaptability in crossbreeding with commercial breeds, making the offspring more efficient conditions (Egito et al., 2002; McManus et al., 2012). In addition, it conserves and promotes the potential and diversified use of Brazilian breeds, avoiding extinction and maintaining production in adverse climate conditions (Egito et al., 2002; McManus et al., 2012).

Given that drug use and vector controls are difficult strategies to implement, and the environmental and food security impacts of these strategies are negative, one of the most valuable features of disease control is exploiting local breeds that are tolerant to infection. Examples are West African N'Dama cattle, which are tolerant to trypanosomosis, and East African Red Maasai sheep, which are resistant to gastrointestinal worms. Adding to the problem are the lack of resources of low-income cattle breeders, which hamper access to treatment and veterinary services, as well as the resistance of pathogens against the inappropriate use of antimicrobials. In this scenario, genetic management of animals is an alternative to increase resistance to diseases by using the most appropriate breeds for each type of production and improving herds by choosing animals with an increased level of resistance, in addition to crossbreeding to incorporate resistance genes in breeds lacking them (FAO, 2007).

The aim of this study was to analyze the diversity and population structure of CPD cattle by evaluating microsatellite markers (STR) of breed characterization. Additionally, STR were associated to animals serological profile of brucellosis, leptospirosis, leucosis, infectious bovine rhinotracheitis (IBR), bovine viral diarrhea (BVD), and neosporosis.

## Material and Methods

### Sampling and Serology

A total of 1,100 blood samples collected in 2011 from male and female CPD cattle of different age groups were used. The samples were obtained from the project “Central-West Pro Network—Characterization, Conservation, and Use of Local Brazilian Cattle Races: Curraleiro and Pantaneiro,” which also maintains data on the serological profile of herds in the states of Piauí, Tocantins, and Goiás.

The epidemiological profile data were determined by serological results (positive/negative/suspect) and responses to epidemiological questionnaires applied at the time of sample collection. The serological tests and places where they were performed as follows: serum agglutination test with buffered acidified antigen (AAT Tecpar^®^ Brazil) to detect anti-*Brucella abortus* antibodies (Brasil. Ministério da Agricultura Pecuária e Abastecimento, 2006), performed in the Graduate Multi-purpose Laboratory of the School of Veterinary and Animal Sciences of the Federal University of Goiás (UFG) (EVZ/UFG); indirect enzyme-linked immunosorbent assay (IDEXX Laboratories, Inc.) to determine antibodies against leukosis, IBR, and BVD viruses according to World Organisation for Animal Health recommendations (OIE. World Health Organization, 2015; OIE. World Health Organization, 2017; OIE. World Health Organization, 2018), performed at the Laboratory of Animal Virology of the Institute of Tropical Pathology and Public Health, UFG; microscopic agglutination test (OIE. World Health Organization, 2014) to detect antibodies against 19 serovars of *Leptospira* spp., using a cutoff of 1:100, performed in the Leptospirosis Laboratory, EVZ/UFG; indirect immunofluorescence for the detection of antibodies against *Neospora caninum* tachyzoites of the Nc-1 strain, with a cutoff of 1:100 (Alvarez-García et al., 2002), performed in the Protozoology Laboratory of the Veterinary Parasitology Center (CPV), EVZ/UFG.

### Genetic Analysis

Total DNA extraction was performed at the Genetics and Biodiversity Laboratory, UFG, from frozen blood samples. The DNA extraction protocol was adapted from Miller et al. (1988).

The short tandem repeat microsatellites were selected from the list of markers recommended by the Food and Agriculture Organization (FAO) and the International Society for Animal Genetics (ISAG) for studying genetic diversity in cattle. Table 1 shows the 28 microsatellites analyzed with the direct and reverse sequences of the primers, the size range of the amplified fragments in base pairs, and their references.

**TABLE 1:** Microsatellites used in the breed characterization of Curraleiro Pé-Duro.

Micros	BTA	Primer forward	Reverse primer	Interval (pb)	Ref
*BM1314*	26	TTC​CTC​CTC​TTC​TCT​CCA​AAC	ATC​TCA​AAC​GCC​AGT​GTG​G	143–167	1
*BM1818*	23	AGC​TGG​GAA​TAT​AAC​CAA​AGG	AGT​GGC​TTT​TTC​AAG​GTC​CAT​GC	248–278	1
*BM1824*	1	GAG​CAA​GGT​GTT​TTT​CCA​ATC	CAT​TCT​CCA​ACT​GCT​TCC​TTG	176–197	1
*BM2113*	2	GCT​GCC​TTC​TAC​CAA​ATA​CCC	CTT​CCT​GAG​AGA​AGC​AAC​ACA​CC	122–156	1
*BM8125*	17	CTC​TAT​CTG​TGG​AAA​AGG​TGG​G	GGG​GGT​TAG​ACT​TCA​ACA​TAC​G	109–123	1
*CRSM60*	10	AAG​ATG​TGA​TCC​AAG​AGA​GGC​A	AGG​ACC​AGA​TCG​TGA​AAG​GCA​TAG	79–115	2
*CSSM66*	14	ACA​CAA​ATC​CTT​TCT​GCC​AGC​TGA	AAT​TTA​ATG​CAC​TGA​GGC​TTG​G	171–209	3
*ETH10*	5	GTT​CAG​GAC​TGG​CCC​TGC​TGC​TAA​CAA​CAA​CA	CCT​CCA​GCC​CAC​TTT​CTC​TTC​TC	207–231	4
*ETH185*	17	TGC​ATG​GAC​AGA​GCA​GCC​TGG​C	GCA​CCC​CAA​CGA​AAG​CTC​CCA​G	214–246	5
*ETH225*	9	GAT​CAC​CTT​GCC​ACT​ATT​TCC​T	ACA​TGA​CAG​CCA​GCT​GCT​ACT​ACT	131–159	5
*ETH3*	19	GAA​CCT​GCC​TCT​CCT​GCA​TTG​G	ACT​CTG​CCT​GTG​GCC​AAG​TAG​G	103–133	4
*HAUT24*	22	CTC​TCT​CTG​CCT​TTT​TGT​CCC​TGT	AAT​ACA​CTT​TAG​GAG​AAA​AAT​A	104–158	6
*HAUT27*	27	TTT​TAT​GTT​CAT​TTT​TTG​ACT​GG	AAC​TGC​TGA​AAT​CTC​CAT​CTT​A	120–158	6
*HEL13*	11	TAA​GGA​CTT​GAG​ATA​AGG​AG	CCA​TCT​ACC​TCC​ATC​TTA​AC	178–200	7
*HEL9*	8	CCCATTCAGTCTTCAGGT	CAC​ATC​CAT​GTT​CTC​ACC​AC	141–173	7
*ILSTS11*	14	GCT​TGC​TAC​ATG​GAA​AGT​GC	CTA​AAA​TGC​AGA​GCC​CTC​TAC​C	261–271	8
*ILSTS6*	7	TGT​CTG​TAT​TTC​TGC​TGT​GG	ACA​CGG​AAG​CGA​TCT​AAA​CG	277–309	9
*INRA23*	3	GAG​TAG​AGC​TAC​AAG​ATA​AAC​TTC	TAA​CTA​CAG​GGT​GTT​AGA​TGA​ACT​C	195–225	10
*INRA32*	11	AAA​CTG​TAT​TCT​CTA​ATA​GCT​AC	GCA​AGA​CAT​ATC​TCC​ATT​CCT​TTT​TTT	160–204	10
*INRA35*	16	ATC​CTT​TGC​AGC​CTC​CAC​ATT​G	TTG​TGC​TTT​ATG​ACA​CTA​TCC​G	100–124	10
*INRA37*	10	GAT​CCT​GCT​TAT​ATT​TAA​CCA​CAC	AAA​ATT​CCA​TGG​AGA​GAG​AAA​C	112–148	10
*INRA63*	18	ATT​TGC​ACA​AGC​TAA​ATC​TAA​CC	AAA​CCA​CAC​AGA​AAT​GCT​TGG​AAG	167–189	10
*MM12*	9	CAA​GAC​AGG​TGT​TTC​AAT​CT	ATC​GGA​CTC​TGG​GGA​TGA​TGT	101–145	11
*SPS115*	15	AAA​GTG​ACA​CAA​CAG​CTT​CTC​CAG	AAC​GAG​TGT​CCT​AGT​TTG​GCT​GTG	234–258	2
*TGLA122*	21	CCC​TCC​TCC​AGG​TAA​ATC​AGC	AAT​CAC​ATG​GCA​AAT​AAG​TAC​ATA​C	136–184	3
*TGLA227*	18	CGA​ATT​CCA​AAT​CTG​TTA​ATT​TGC​T	ACA​GAC​AGA​AAC​TCA​ATG​AAA​GCA	75–105	12
*TGLA53*	16	GCT​TTC​AGA​AAT​AGT​TTG​CAT​TCA	ATC​TTC​ACA​TGA​TAT​TAC​AGC​AGA	143–191	12
*TGLA126*	20	CTA​ATT​TAG​AAT​GAG​AGG​CTT​CTT​CT	TTG​GTC​TCT​ATT​CTC​TCT​GAA​TAT​TCC	115–131	13

Micros., microsatellite name; BTA, chromosome location; forward and reverse primers, primer sequence; bp, base pairs; Ref, literature reference. 1 (Bishop et al*.* 1994); 2 (Moore et al., 1994); 3 (Barendse et al*.* 1994); 4 (Toldo et al., 1993); 5 (Steffen et al., 1993); 6 (Thieven et al., 1997); 7 (Kaukinen and Varvio 1993); 8 (Brezinsky et al., 1993a); 9 (Brezinsky et al., 1993b); 10 (Vaiman et al., 1994); 11 (Mommens et al., 1994); 12 (Kappes et al., 1997).

Source: Adapted from Queiroz (2007) and FAO (2011).

Polymerase chain reaction (PCR) was prepared and the PCR was performed as recommended with 2 µL of DNA, 3 µL of primer mix (0.2–0.4 µM of each primer), and 5 µL of commercial mix (Taq PCR Master Mix Kit, QIAGEN^®^, United States).

Primer amplification was optimized *in-house* by testing the optimal fragment amplification temperatures for multiplex reactions. To standardize the reactions, two positive samples previously genotyped were tested in multiplex PCRs with a temperature gradient between 55 and 62°C. The samples were grouped by amplified fragment size and fluorochrome used to avoid fragment overlapping. The primers from each electropherogram were divided into two multiplex PCRs (M1 and M2), one with an annealing temperature of 55°C and the other with an annealing temperature of 62°C, chosen based on the best amplification signal in each reaction.

The first and second multiplex PCRs of the first electropherogram and the second multiplex PCR of the second electropherogram (Gel1M1, Gel1M2, and Gel2M2) followed the amplification protocol of an initial cycle at 95°C for 5 min; 34 amplification cycles (95°C for 40 s, 55°C for 50 s, and 72°C for 1 min); final extension at 72°C for 30 min, followed by an infinite temperature of 12°C. PCR of the Gel2M1 primers was performed with an initial cycle at 95°C for 5 min; 34 cycles (95°C for 30 s and 62°C for 4 min), final extension at 60°C for 20 min, and infinite temperature of 12°C.

The amplification products of microsatellite loci were genotyped in capillary electrophoresis systems using the ABI3500 automatic sequencer (Applied Biosystems^®^) at the Replicon/LaGene Research Center of the Pontifical Catholic University of Goiás and the ABI 3130 automatic sequencer (Applied Biosystems^®^) at the University of Córdoba, Spain. The data were analyzed using the GeneMapper program (v4.0, Applied Biosystems™). The size of the amplified fragments was defined in comparison with the standard Gene Scan LIZ 600^®^ (Life Technologies) and two control samples in each electropherogram. The controls were samples previously genotyped by the company Animal Breeding Consulting, Spain, with known and standardized genotypes.

Filtering of the genotypes was performed to remove samples that failed to amplify half or more of the markers because more genotyping failures mean that more assignment errors can occur. The P6 property was excluded as it had no DNA samples for the genetic analyses. At the end of the quality control, 608 remaining samples were evaluated together, without prior population definition (Table 2). The dataset can be consulted online (https://figshare.com/account/projects/98876/articles/14069039).

**TABLE 2:** Collection state, sampled properties, city, number of DNA samples processed, and final number of samples analyzed after filtering of genotypic data.

State	Property	Municipality	DNA samples genotyped and evaluated
Tocantins	T1	1-Guarai	44
	T2	2-Porto Nacional	25
	T3	3-Sucupira	14
	T4	4-Chapada Natividade	10
Piauí	P1	5-Teresina	82
	P2	6-Campo Maior	20
	P3	6-Campo Maior	19
	P4	7-Palmeiras	16
	P5	8-Oeiras	19
	P7	9-Elesbão Veloso	20
	P8	6-Campo Maior	5
	P9	6-Campo Maior	11
Goiás	G1	10-Cavalcante	73
	G2	11-Monte Alegre	21
	G3	12-Planaltina	39
	G4	13-Campestre	84
	G5	14-Mimoso de Goiás	53
	G6	15-Pilar de Goiás	28
	G8	17-Pirenópolis	25
TOTAL: 3	19	17	608

### Statistical Analysis

The data obtained were treated in order to eliminate samples with low genotyping quality and monomorphic markers, leaving 608 Curraleiro Pé Duro samples that showed more than 50% of the genotyped loci and, therefore, were selected for the association analyses (Table 2). The dataset can be consulted online (https://figshare.com/account/projects/98876/articles/14069039).

The genotyping data were entered into Structure 2.3.4 software (Pritchard et al., 2000), and the samples were grouped according to the similarity of multilocus genotypes. The program assigned the individuals to the clusters of greatest similarity, determining the k value, that is, the number of populations in which the samples were divided based on Bayesian statistics through the Markov Chains Monte Carlo (MCMC) method (Pritchard et al., 2000).

To estimate population structure in Structure software, runs were conducted using a parameter set of 10,000 burn-in generations followed by 500,000 MCMC iterations. One to 22 populations (k = 1 to k = 22) and five iterations for each k value were assumed in the admixture model (Haikukutu 2018). The results of the cluster determination analyses were processed, and the real k value was determined using the ΔK method proposed by Evanno et al. (2005) using the Structure Harvester software (Earl and vonHoldt, 2012). The k value that best accommodated the allelic diversity, which corresponds to the stabilization point of the curve, was observed in graphical analysis.

The genetic diversity indicators were calculated in the Microsatellite Toolkit for Excel program (Park 2001). The allele frequencies, number of alleles per locus, allele richness, observed and expected heterozygosity, and polymorphic information content (PIC) were obtained (Park 2001). PIC values were used to evaluate the quality of the markers (Botstein et al., 1980) as follows: above 0.5, very informative; from 0.25 to 0.5, moderately informative; below 0.25, poorly informative (McManus et al., 2011).

The deviations in Hardy–Weinberg equilibrium (HWE) were estimated with the software Genepop 4.0.5.3 (Raymond and Rousset 1995) by applying the MCMC method. In the Populations 1.2.28 program (Langella 1999), genetic distance matrices were built using the methodology of Reynolds and Nei’s Da (Nei et al., 1983) and the respective phylogenetic trees. The tree of genetic distances constructed using the neighbor-joining method was graphically plotted using Mega 7.0.26 software (Kumar et al., 2016) to observe associations among populations. Correspondence factor analysis (CFA) was performed in Genetix software (Belkhir et al., 2004) to determine the divergence of the CPD populations.

The F coefficients, namely, Wright’s fixation index (F), which includes Fis (inbreeding coefficient/heterozygosity deficiency coefficient), Fit (inbreeding or fixation index for the total population), and Fst (fixation index in the subpopulation or estimate of genetic differentiation between subpopulations) (McManus et al., 2011) were calculated in Genetix. The method of Weir and Cockerham (1984) was used, estimating a 95% confidence interval with 1,000 bootstraps per locus.

Genetic divergence among populations was assessed based on analysis of molecular variance (AMOVA) (Excoffier et al., 1992) in Arlequin software version 3.5.2.2 (Excoffier and Lischer 2010), using pairwise Fst genetic distances as a method to calculate intra- and inter-group genetic distances.

### Association Between Breed Molecular Markers and Disease

Breed characterization markers were associated with the frequency of animals seropositive against infections by pathogens causing brucellosis, leptospirosis, neosporosis, leukosis, IBR, and BVD.

A population study was carried out adopting each genetic cluster as a population. The allele frequencies were observed and compared between groups of sick (positive) and healthy individuals (negative) using Fisher’s exact test. The strength of association was measured using the relative risk (*odds ratio*), which indicates how frequently a disease occurs in an individual carrying a certain marker.

## Results

CPD cattle were assigned to clusters in order to observe subpopulations within the breed, and the results of the first analysis showed that the most appropriate grouping was k = 3.

**FIGURE 1:**
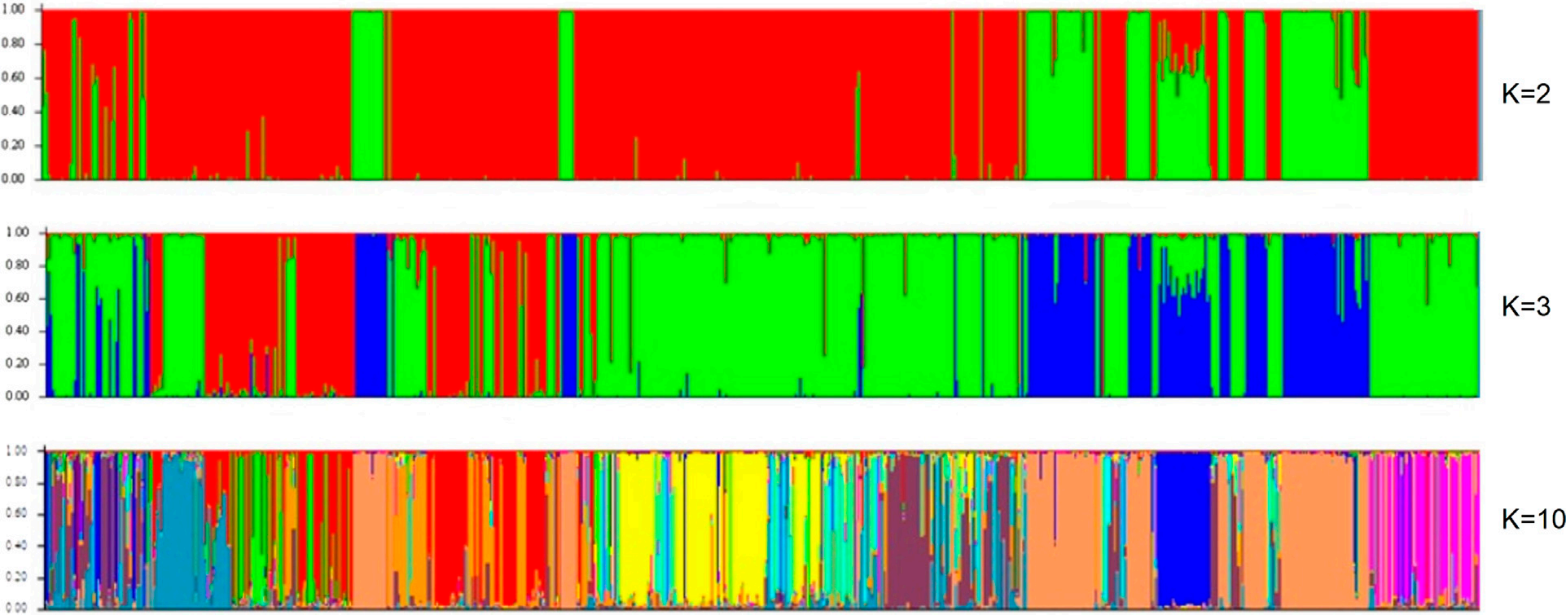
Populations of Curraleiro Pé-Duro cattle detected by the method of clusters with genetic similarity using breed characterization markers.

Figure 1 depicts graphs in which each individual corresponds to a vertical line divided into k genotype segments, according to the number of inferred clusters. Each cluster is represented by a color, and individuals whose genotype is classified in more than one cluster have more than one color, indicating the occurrence of an admixture.

**FIGURE 2:**
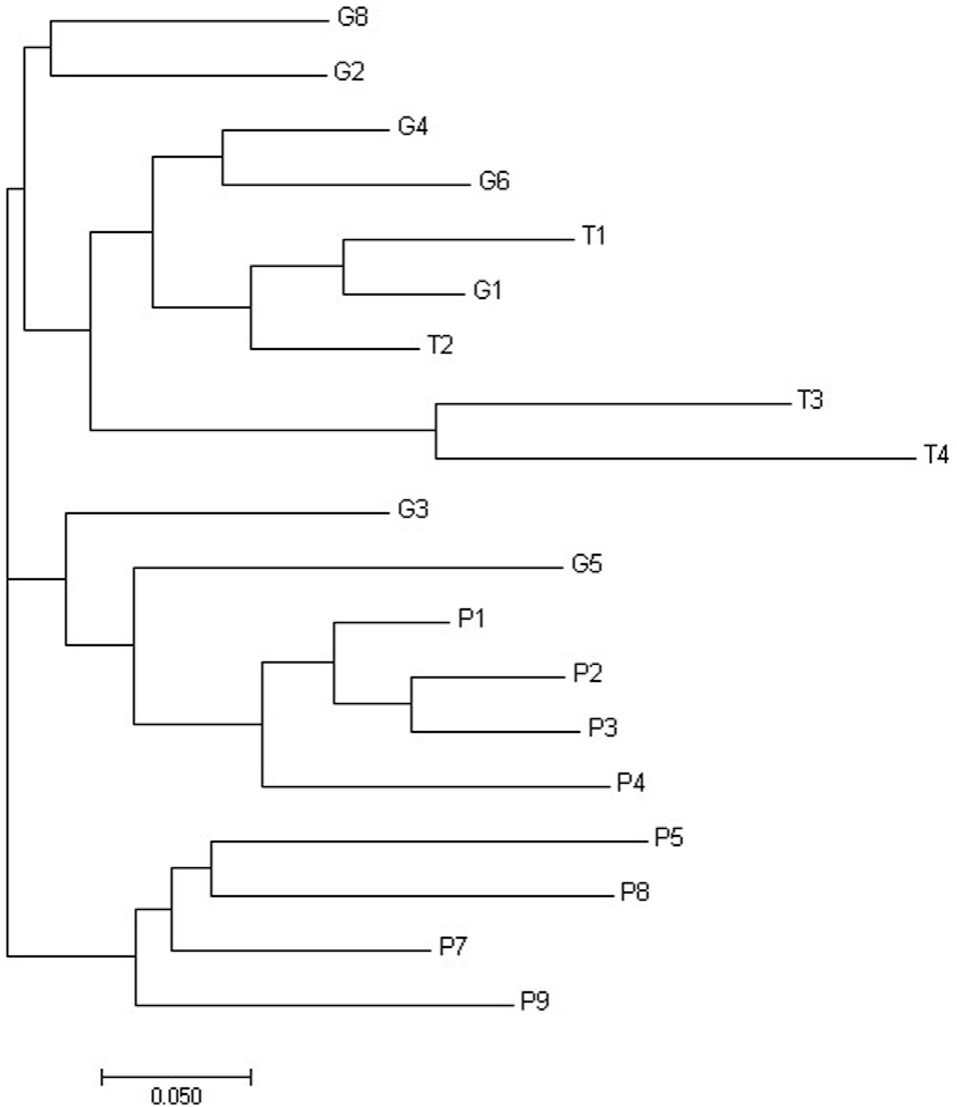
Phylogenetic tree of Da distances of Nei built using the neighbor-joining method for the properties of Curraleiro Pé-Duro cattle.

Figure 2 shows threshold of significant difference between CPD populations, that were best clustering in three groups.

The populations and the number of individuals in each population assigned to clusters are shown in Table 3. Populations of Piauí have most of the genotypes allocated in cluster 1, while populations of Tocantins are mostly grouped in cluster 3. The properties of the state of Goiás had genotypes grouped in all clusters, indicating the absence of clear differentiation between the alleles present in individuals from this state and those of others.

**TABLE 3:** Locality of cattle assigned to clusters 1, 2, and 3 of breed genetic similarity for the Curraleiro Pé-Duro breed.

State	Rural properties	Proportions of individual (Number of individuals in Cluster/Total of individuals)		
		Cluster 1	Cluster 2	Cluster 3
GO	G1	26.02% (19/73)	0% (0/73)	7.97% (54/73)
	G2	71.43% (15/21)	0% (0/21)	28.57% (6/21)
	G3	48.72% (19/39)	46.15% (18/39)	5.13% (2/39)
	G4	20.24% (17/84)	60.71% (51/84)	19.05% (16/84)
	G5	35.85% (19/53)	64.15% (34/53)	0% (0/53)
	G6	32.14% (9/28)	42.86% (12/28)	25% (7/28)
	G8	72% (18/25)	0% (0/25)	28% (7/25)
PI	P1	93.90% (77/82)	6.09% (5/82)	0% (0/82)
	P2	100% (20/20)	0% (0/20)	0% (0/20)
	P3	94.74% (18/19)	5.26% (1/19)	0% (0/19)
	P4	81.25% (13/16)	6.25% (1/16)	12.5% (2/16)
	P5	100% (19/19)	0% (0/19)	0% (0/19)
	P7	95% (19/20)	0% (0/20)	5% (1/20)
	P8	100% (5/5)	0% (0/5)	0% (0/5)
	P9	95% (10/11)	0% (0/11)	9.10% (1/11)
TO	T1	20.45% (9/44)	4.54% (2/44)	75% (33/44)
	T2	56% (14/25)	0% (0/25)	44% (11/25)
	T3	7.14% (1/14)	0% (0/14)	92.86% (13/14)
	T4	0% (0/10)	0% (0/10)	100% (10/10)
**Total number of individuals/cluster**		**321**	**124**	**163**	

**FIGURE 3:**
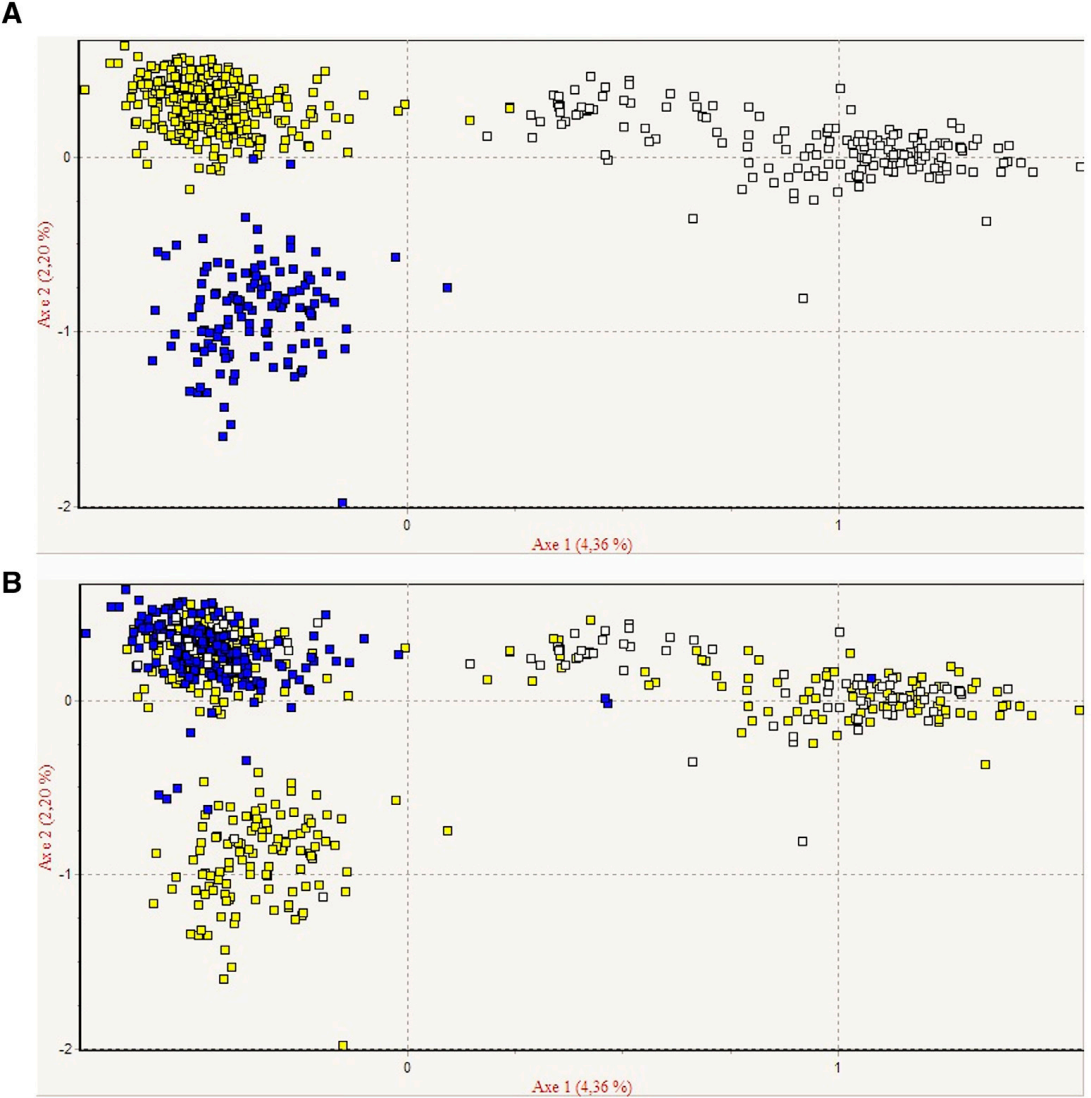
Graphical representation of the factor analysis of correspondence of Curraleiro Pé-Duro cattle **(A)**. Representation by clusters of genetic similarity (cluster 1 in yellow, cluster 2 in blue, and cluster 3 in white) **(B)**. Representation by state (Goiás in yellow, Piauí in blue, and Tocantins in white).

The genetic distances between populations were calculated according to Nei’s Da, and the phylogenetic tree was built using the neighbor-joining method (Figure 3). The grouping by genetic distances showed small differences in comparison to the grouping by Structure software. It is possible to observe three clusters, separating the properties of Tocantins from those of Piauí and the properties of Goiás among the properties of other states.

The genetic diversity indices, described in Table 4, were measured by estimating the levels of expected and observed heterozygosity and the average number of alleles. The expected heterozygosity was higher than that observed in the three clusters, which, however, had high allele richness.

**TABLE 4:** Indices of genetic diversity among clusters of breed characterization of Curraleiro Pé-Duro cattle.

Population	AM	Loci	He	Ho	NMA	SD NMA	Total alleles
*Cluster* 1	321	28	0.7565	0.6398	10.54	3.45	295
*Cluster* 2	124	28	0.7315	0.5670	8.32	2.63	233
*Cluster* 3	163	28	0.7677	0.6330	10.82	2.48	303

AM, number of samples; He, expected heterozygosity; H0, observed heterozygosity; NMA, mean number of alleles per loci; SD NMA, standard deviation of the mean number of alleles.

Higher presence of alleles was observed in breed clusters 2 and 3 (233 alleles in 124 samples and 303 alleles in 163 samples analyzed, respectively) than in cluster 1 (295 alleles in 321 samples analyzed). The markers used were tested using HWE, and all loci in cluster 3 were in disequilibrium (*p* < 0.05). In clusters 1 and 2, some markers were in equilibrium, i.e., no decrease in heterozygotes relative to homozygotes was observed at these loci. The PIC values per marker are compiled in Table 5.

**TABLE 5:** Heterozygosity of microsatellite loci for breed characterization of Curraleiro Pé-Duro cattle.

Loci	Cluster 1				Cluster 2				Cluster 3			
	HWE	Ho	He	PIC	HWE	Ho	He	PIC	HWE	Ho	He	PIC
*BM1314*	**	0.551	0.66	0.61	**	0.47	0.61	0.54	**	0.59	0.71	0.67
*BM1818*	0.062	0.72	0.719	0.67	**	0.388	0.563	0.471	**	0.635	0.719	0.685
*BM1824*	0.244	0.698	0.738	0.69	*	0.678	0.73	0.689	**	0.755	0.825	0.799
*BM8125*	0.586	0.769	0.765	0.73	**	0.804	0.744	0.706	**	0.861	0.812	0.792
*CSSM66*	0.773	0.79	0.83	0.8	**	0.652	0.777	0.739	**	0.727	0.855	0.838
*ETH10*	0.512	0.659	0.659	0.61	0.114	0.58	0.658	0.592	**	0.538	0.718	0.675
*ILSTS11*	**	0.581	0.612	0.54	**	0.604	0.724	0.679	**	0.57	0.786	0.75
*INRA32*	1	1	0.933	0.74	**	0.642	0.801	0.768	**	0.755	0.815	0.792
*INRA35*	**	0.369	0.473	0.41	**	0.51	0.681	0.624	**	0.287	0.65	0.602
*INRA37*	**	0.684	0.807	0.78	0.056	0.613	0.692	0.65	**	0.682	0.772	0.739
*MM12*	0.315	0.747	0.781	0.74	**	0.617	0.812	0.783	**	0.726	0.855	0.836
*TGLA122*	*	0.819	0.813	0.79	1	1	0.833	0.554	**	0.694	0.875	0.86
*TGLA126*	**	0.55	0.666	0.61	0.191	0.686	0.604	0.552	**	0.668	0.775	0.739
*BM2113*	**	0.575	0.881	0.86	**	0.351	0.772	0.737	**	0.5	0.784	0.761
*CSRM6O*	**	0.689	0.723	0.69	**	0.639	0.761	0.722	**	0.702	0.822	0.798
*ETH185*	**	0.546	0.797	0.76	**	0.581	0.764	0.73	**	0.54	0.805	0.778
*ETH225*	**	0.543	0.806	0.77	**	0.405	0.821	0.792	**	0.695	0.824	0.799
*ETH3*	**	0.729	0.809	0.78	*	0.71	0.757	0.72	**	0.759	0.787	0.755
*HAUT24*	**	0.351	0.839	0.81	**	0.505	0.82	0.795	**	0.564	0.824	0.805
*HAUT27*	**	0.678	0.844	0.82	**	0.509	0.774	0.748	**	0.265	0.608	0.5824
*HEL13*	**	0.545	0.67	0.62	**	0.603	0.724	0.68	**	0.634	0.772	0.7402
*HEL9*	**	0.753	0.76	0.73	**	0.846	0.714	0.663	**	0.792	0.845	0.825
*ILSTS6*	**	0.481	0.77	0.74	**	0.453	0.794	0.762	**	0.549	0.804	0.773
*INRA23*	**	0.493	0.68	0.65	**	0.052	0.618	0.544	*	0.645	0.666	0.63
*INRA63*	**	0.671	0.67	0.61	0.066	0.333	0.866	0.671	**	0.919	0.529	0.417
*SPS115*	**	0.625	0.7	0.66	**	0.623	0.727	0.693	*	0.583	0.681	0.645
*TGLA227*	**	0.699	0.88	0.86	**	0.825	0.866	0.848	**	0.807	0.852	0.833
*TGLA53*	**	0.581	0.85	0.83	**	0.184	0.457	0.438	**	0.267	0.706	0.676

HWE, Hardy–Weinberg equilibrium. *Hardy–Weinberg equilibrium marker *p* < 0.05 and **p < 0.01; Ho, observed heterozygosity; He, expected heterozygosity; PIC, polymorphic information content.

All microsatellite markers evaluated were highly polymorphic and highly informative as they had PIC values above 0.5, with few markers with PIC values above 0.4. Although highly informative, the heterozygosity observed was lower than expected in most loci, suggesting increased homozygosity. The Fis, Fit, and Fst were calculated per marker and per cluster and are shown in Table 6.

**TABLE 6:** Results of F statistical analysis by phylogenetic clusters and by loci in the Curraleiro Pé-Duro cattle populations.

Marker	Cluster 1			Cluster 2			Cluster 3			Fis by marker
	Fis	Fit	Fst	Fis	Fit	Fst	Fis	Fit	Fst	
*BM1314*	0.1927	0.2545	0.0766	0.1763	0.267	0.1101	0.1867	0.2092	0.0277	0.1608
*BM1818*	0.1478	0.2128	0.0762	0.0369	0.1405	0.1076	0.0247	0.0688	0.0452	0.1653
*BM1824*	0.0798	0.1132	0.0363	0.0652	0.0957	0.0327	0.0588	0.0806	0.0232	0.1651
*BM2113*	0.4081	0.4292	0.0355	0.3526	0.4059	0.0823	0.3723	0.4344	0.0989	0.1528
*BM8125*	−0.068	0.0258	0.0886	−0.032	0.0639	0.093	−0.035	−0.022	0.0133	0.1694
*CSRM6O*	0.1523	0.2666	0.1348	0.0825	0.2072	0.1359	0.0792	0.102	0.0248	0.1639
*CSSM66*	0.1538	0.2697	0.1369	0.1044	0.1946	0.1007	0.0906	0.215	0.1367	0.1634
*ETH10*	0.2088	0.4085	0.2524	0.0872	0.3176	0.2525	0.024	0.0527	0.0294	0.1639
*ETH185*	0.2928	0.3974	0.1479	0.3202	0.4131	0.1367	0.2951	0.299	0.0055	0.1559
*ETH225*	0.2996	0.3741	0.1064	0.2632	0.336	0.0988	0.3792	0.384	0.0077	0.1554
*ETH3*	0.0467	0.076	0.0307	0.0782	0.088	0.0106	0.0891	0.1131	0.0264	0.1649
*HAUT24*	0.3446	0.4159	0.1088	0.474	0.5369	0.1196	0.5181	0.5716	0.111	0.1495
*HAUT27*	0.4509	0.5891	0.2517	0.3085	0.4217	0.1637	0.2429	0.3031	0.0795	0.1557
*HEL13*	0.1737	0.2809	0.1297	0.1827	0.3338	0.185	0.1784	0.1919	0.0165	0.1609
*HEL9*	−0.029	0.1061	0.1319	0.0358	0.1263	0.0939	−0.035	−0.011	0.0229	0.1679
*ILSTS11*	0.2338	0.3098	0.0992	0.1275	0.285	0.1805	0.0746	0.1206	0.0497	0.1624
*ILSTS6*	0.3512	0.4065	0.0853	0.344	0.4132	0.1054	0.4005	0.4321	0.0527	0.1537
*INRA23*	0.1234	0.4087	0.3255	0.1169	0.3801	0.2981	0.3941	0.4662	0.119	0.1612
*INRA32*	0.1272	0.1551	0.032	0.0688	0.0427	−0.027	0.1896	0.1406	−0.06	0.163
*INRA35*	0.4324	0.5632	0.2304	0.3519	0.5464	0.3001	0.2304	0.4219	0.2488	0.1569
*INRA37*	0.1161	0.1499	0.0382	0.1394	0.152	0.0146	0.1411	0.1636	0.0262	0.1626
*INRA63*	−0.704	−0.019	0.402	−0.461	0.1135	0.3936	0.0356	0.1509	0.1195	0.1786
*MM12*	0.1884	0.2997	0.1372	0.0827	0.1527	0.0764	0.0993	0.2599	0.1783	0.1634
*SPS115*	0.1441	0.3565	0.2481	0.1212	0.3498	0.2601	0.1205	0.1291	0.0098	0.1628
*TGLA122*	0.2031	0.2482	0.0566	0.0681	0.1626	0.1014	−0.009	0.1007	0.1087	0.1654
*TGLA126*	0.0328	0.1624	0.134	0.1618	0.2523	0.108	0.0962	0.1521	0.0618	0.1633
*TGLA227*	0.051	0.1135	0.0659	0.1525	0.2117	0.0699	0.1603	0.1682	0.0095	0.1629
*TGLA53*	0.6133	0.7663	0.3957	0.4127	0.5263	0.1935	0.3805	0.557	0.2848	0.1516

The results indicated similar values between clusters but differences for each marker. The Fis (fixation index within population) per marker, considering all animals independently of clusters, was similar for all loci. Fis values ranged between −0.70401 (INRA63) and 0.6136 (TGLA53) in cluster 1; 0.46182 (INRA63) and 0.47398 (HAUT24) in cluster 2; and −0.03575 (BM8125) and 0.51811 (HAUT24) in cluster 3. The negative Fis values in some loci indicated that the observed heterozygosity was greater than expected, with no reduction of heterozygotes in comparison with homozygotes.

Fst values ranged between 0.03073 (ETH3) and 0.402 (INRA63) in cluster 1, between 0.00 (INRA32) and 0.39359 (INRA63) in cluster 2, and between 0.00 (INRA32) and 0.28483 (TGLA53) in cluster 3, indicating varying levels of differentiation from markers with little genetic differentiation (Fst 0.25). Fst levels close to zero indicated less genetic differentiation.

CFA (Figure 3A) graphically represented three genetic groups. The same analysis was performed considering the separation by state to observe how the clusters were formed (Figure 3B). Cluster one consisted of herds from the states of Piauí, cluster two of herds from Goiás, and cluster three of herds from Tocantins and Goiás.

The frequencies of infections in the three genetic clusters were compared to the frequencies of diseases in clusters defined by environmental characteristics, according to a study using the same samples (Lobo 2018). Chi-squared analysis, performed to detect associations between genetic and environmental clusters, showed a significant difference (*p* < 2.2e-16), with 53.45% (325/608) of concordant samples. Moreover, the properties grouped according to environmental characteristics were also grouped according to genetic characteristics (Table 7).

**TABLE 7:** Frequency distribution of seropositive individuals against infections according to the classification by genetic and environmental clusters.

*Clusters*	BRU	LPT	NEO	Read	IBR	BVD	n
*cluster1_genetic*	0.31% (1/321)	41.32% (131/317)	48.12% (154/320)	18.29% (58/317)	64.58% (206/319)	44.97% (143/318)	321
*cluster2_genetic*	2.41% (3/124)	45.08% (55/122)	39.34% (48/122)	9.84% (12/122)	66.67% (82/123)	42.5% (51/130)	124
*cluster3_genetic*	0% (0/163)	57.80% (85/161)	20% (32/160)	24.84% (39/157)	68.94% (111/161)	41.83% (64/153)	163
*cluster1_environment*	0% (0/285)	43.3% (123/284)	44.9% (128/285)	23.9% (68/284)	68.1% (194/285)	49.1% (140/285)	285
*cluster2_* environment	1.35% (1/74)	28.8% (21/73)	52.7% (39/74)	0.04% (3/73)	49.3% (36/73)	20.5% (15/73)	74
*cluster3_* environment	1.2% (3/249)	52.3% (127/243)	27.6% (67/243)	15.9% (38/239)	69.0% (169/245)	44.2% (103/233)	249

BRU, brucellosis; LPT, leptospirosis; NEO, neosporosis; LEU, leucosis; IBR, infectious bovine rhinotracheitis; BVD, bovine viral diarrhea; n, number of animals.

A significant difference (*p* < 0.001, Kappa = 0.247) was found between genetic and environmental clusters, with 53.45% of samples in agreement (grouped in genetic and environmental clusters 1, 2, or 3). The percentages of individuals allocated to each cluster in the positive, negative, and suspect groups for each infection were tabulated (Table 8), considering only the genetic clusters.

**TABLE 8:** Prevalence of antibodies against microorganisms that cause brucellosis, leptospirosis, neosporosis, leukosis, rhinotracheitis, and viral diarrhea in Curraleiro Pé-Duro cattle by genetic clusters.

Infection	Result	*Cluster* 1	*Cluster* 2	*Cluster* 3	*p* (Fisher)
BRU	Positive	0.31% (1/321)	2.42% (3/124)	0.0% (0/163)	0.0489
	Negative	99.68% (320/321)	97.58% (121/124)	100% (163/163)	
LPT	Positive	41.32% (131/317)	45.08% (55/122)	52.80% (85/161)	Ns
	Negative	58.67% (186/317)	54.92% (67/122)	47.20% (76/161)	
NEO	Positive	48.13% (154/320)	39.34% (48/122)	20.0% (32/160)	<0.001
	Negative	51.87% (166/320)	60.66% (74/122)	80.0% (128/160)	
READ	Positive	18.30% (58/317)	9.84% (12/122)	24.84% (39/157)	0.00728
	Negative	81.39% (258/317)	90.16% (110/122)	75.16% (118/157)	
	Suspect	0.31% (1/317)	0.0% (0/122)	0.0% (0/157)	
IBR	Positive	64.58% (206/319)	66.67% (82/123)	68.94% (111/161)	Ns
	Negative	33.54% (107/319)	32.52% (40/123)	26.71% (43/161)	
	Suspect	1.88% (6/319)	0.81% (1/123)	4.35% (7/161)	
BVD	Positive	44.97% (143/318)	42.50% (51/120)	41.83% (64/153)	Ns
	Negative	49.37% (154/318)	50.00% (60/120)	48.36% (74/153)	
	Suspect	6.60% (21/318)	7.50% (9/120)	9.80% (15/153)	

BRU, *brucella abortus*; LPT, *Leptospira* sp.; NEO, *neospora caninum*; LEU, enzootic bovine leukosis; IBR, infectious bovine rhinotracheitis; BVD, bovine viral diarrhea; ns, not significant (*p* > 0.05).

Cluster 1 had the lowest number of animals positive for *Leptospira* and IBR. Cluster 3 had a lower frequency of positives against *Brucella* and *Neospora*, and cluster 2 had a lower frequency of positives for bovine leukosis, than other clusters. The prevalence by cluster was similar for all infections.

AMOVA considered three groups referring to Goiás, Piauí, and Tocantins states to estimate the amount of total diversity due to variation in populations between and within states (Table 9). The AMOVA provided overall Fit estimate of 0.24491 (*p* < 0.00001, with 20,000 permutations), meaning that the total variation found among populations was 24.49%. Of this, 5.91% corresponded to the variation in populations between states and 18.57% to the variation between populations of the same state.

**TABLE 9:** Results of the analysis of molecular variance for populations of Curraleiro Pé-Duro from three Brazilian states based on microsatellite data.

Source of variation	Sum of squares	Variance components	Variation (%)
Between states	360.02	0.69	5.92
Between populations/within states	6,233.39	2.16	18.57
Within populations	4,187.00	8.80	75.51
Total	10,780.42	11.65	—

The 19 herds were tested as independent groups by AMOVA. This test indicated that 24.72% of the total variation (Fit) was between herds and 75.51% within individuals. The variation between herds was lower than the variation between individuals of the same herd (Table 10).

**TABLE 10:** Results of the analysis of molecular variance for 19 herds of Curraleiro Pé-Duro based on microsatellite data.

Source of variation	Sum of squares	Variance components	Variation (%)
Between groups	1,030.41	0.90	9.56
Between individuals/within groups	4,898.08	1.43	15.16
Within individuals	3,717.50	7.09	75.28
Total	9,645.99	9.42	—

## Discussion

The differentiation of the same breed into three clusters may be explained by the role of the environment in selection. The physical separation of the populations of Goiás, Tocantins, and Piauí caused reproductive isolation, and adaptation to local environmental conditions may have affected the emergence of new phenotypes (Lofeu and Kohlsdorf 2015). The genetic clusters resembled the clusters defined by environmental characteristics (Lobo 2018), corroborating the association of genotype and environment in the formation of the phenotype, which was expressed by the occurrence of antibodies against diseases.

Expected heterozygosity was higher than observed except for loci BM8125 and INRA63 (cluster 1), which may indicate increased inbreeding and reduced genetic variability. The loss of diversity in breed characteristics may also indicate an increase in inbreeding coefficient.

All loci were polymorphic, with a total of 295 alleles in cluster 1, 233 in cluster 2, and 303 in cluster 3. Mean values of 10.54, 8.32, and 10.82 alleles per locus were observed in clusters 1, 2, and 3, respectively, a value lower than the average of 13 alleles per loci previously described in the breed. Although polymorphic, the loci were in HWE imbalance, which was in agreement with the results of Oliveira (2008) n which HWE deviations were observed in nine of 10 loci analyzed in CPD breed.

The increase in the number of homozygotes, observed by the deviation in the HWE, can be explained by the high level of inbreeding due to the small effective number of animals in reproductive age and mating between related individuals. Other factors that can also explain the deviation in the HWE are subdivisions within the population, natural selection, migration, and null alleles (Quiroz 2007).

The Fst values were lower than 0.15, indicating that the two subpopulations share alleles and, therefore, the genetic differentiation is low to moderate. Owing to the geographical isolation of the populations of the states of Piauí, Tocantins, and Goiás, high differentiation between the populations of the states and increased of inbreeding within each state would be expected because the populations do not mate at random (Oliveira 2008; McManus et al., 2011). However, we estimate that exchanging sires between properties to decrease the effect of inbreeding causes different properties to share alleles, reducing the differentiation between properties. The exchange or purchase of animals of the breed is limited by the number of farms, and the replacement of the herd is conditioned to the availability of sires among the properties studied.

Fis ranged from 0.16441 (TGLA53) to 0.19427 (INRA63) and Fit from 0.27191 (TGLA53) to 0.29383 (BM8125). Fis values indicated inbreeding. The loss of genetic variability in CPD herds is a consequence of inbreeding due to the replacement of sires with animals from their own herd. The CPD breed showed higher F indices than other native Brazilian breeds such as Caracu, Pantaneiro, and Mocho Nacional (Egito 2007; Fioravanti et al., 2011).

The number of positive animals was lower than that of negative animals, except for IBR virus infection in all clusters and for *Leptospira* infection, in cluster 3. The high rate of seropositivity for viral infections in CPD was reported by Juliano (2006) and Amaral (2013), concluding that the viruses are endemically present in the evaluated populations.

It is possible to infer that past environmental changes have triggered phenotypic diversification events that fixed or eliminated alleles (Lofeu and Kohlsdorf 2015) in populations from different geographic regions, influenced by physical distance, causing the three gene clusters to express different disease susceptibility/resistance phenotypes.

In CPD animals, the most advantageous alleles for adaptation to the environmental conditions of Piauí and Tocantins showed increased frequency in each environment and were fixed in the populations, subdividing the breed into three distinct populations. AMOVA findings showed that most of the total genetic variance was due to the difference between alleles within individuals (75.28%), which is a similar result to that described for the local Brazilian breed Crioulo Lageano (Spritze et al., 2003; Carvalho et al., 2012). CPD herds were similarly divided into breed characterization clusters and geographic clusters due to herd isolation. However, since the variation of genotypes was greater among individuals of the same group than between herds or states, individuals from the same property were grouped into different clusters.

## Conclusion

Diversity and a high number of alleles were detected in the three genetic clusters; however, the deviation from the HWE and the inbreeding coefficient indicated the occurrence of inbreeding in the populations. The difference in antibody detection was significant for *Brucella* spp., *N. caninum*, and BLV between clusters. The greatest variation in genotypes related to the *BoLA* genes occurred at the individual level, and has a high number of alleles that provide varied phenotypes to CPD.

We conclude that the populations are in the process of genetic differentiation. However, this differentiation is more pronounced in the Piauí herds compared to Tocantins and Goias. The results were not sufficient to indicate a genetic subpopulation with increased resistance or susceptibility to infection.

## Data Availability

The datasets analysed during the current study are available in https://doi.org/10.6084/m9.figshare.14069039.
